# Light-Controlled Membrane Fusion in Synthetic Cells

**DOI:** 10.3390/life16020317

**Published:** 2026-02-12

**Authors:** Boying Xu, Adriano Caliari, Jian Xu

**Affiliations:** 1 School of Ecological and Environmental Sciences, East China Normal University, 500 Dongchuan Road, Shanghai 200241, China; byxu@des.ecnu.edu.cn; 2 Institute for Bioengineering of Catalonia (IBEC), Baldiri Reixac 10, 08028 Barcelona, Spain; 3 Laboratory of Insect Genome Science, Kyushu University Graduate School of Bioresource and Bioenvironmental Sciences, Motooka 744, Nishi-ku, Fukuoka 819-0395, Japan

**Keywords:** light-induced membrane fusion, synthetic cells, lipid vesicles, optogenetic tools, nanotechnology

## Abstract

Light-induced membrane fusion has become a pivotal technique for constructing and functionalizing synthetic cells by enabling precise control over membrane merging events. Traditional fusion approaches that rely on chemical, physical, and mechanical stimuli frequently lack both specificity and reversibility, limiting their utility in mimicking dynamic cellular processes. Here, we review advances employing photosensitive molecules and optogenetic tools that facilitate spatiotemporally controlled fusion of lipid and polymer vesicles, enabling dynamic content exchange and membrane remodeling. These approaches have enhanced synthetic cell assembly, molecular transport, and signal transduction, with applications extending to drug delivery and biosensing. Despite challenges in efficiency and biocompatibility, ongoing innovations in photosensitizer design and light activation strategies promise to expand the capabilities of synthetic biology platforms. This work underscores the potential of light-induced fusion to advance the development of intelligent nanomaterials and functional synthetic cellular systems.

## 1. Introduction

The design and functionalization of synthetic cells represent a frontier in synthetic biology, with the goal of replicating the complex functionalities of natural cells using engineered artificial systems. Central to this effort is the precise assembly and regulation of membrane structures, as membranes are fundamental interfaces mediating compartmentalization, signaling, molecular transport, and communication [1,2]. Various types of synthetic cells can be constructed from lipid or polymeric vesicles—such as giant unilamellar vesicles (GUVs) and polymersomes—which serve as minimalistic models that recapitulate essential cellular characteristics, including compartmentalization and dynamic biochemical processes [3,4,5].

Engineering membrane fusion/division events within these systems is pivotal, as this process underlies critical operations including intracellular trafficking, cell–cell and organelle communication, and signal transduction [6,7,8,9]. In natural cells, membrane fusion is highly orchestrated by specific protein machinery; for example, SNARE complexes facilitate vesicle docking and fusion, enabling precise cargo delivery and membrane remodeling [10,11]. Replicating such sophisticated processes in synthetic cells requires innovative strategies to achieve controlled and programmable fusion. Traditional fusion techniques that rely on chemical fusogens or electrostatic interactions often lack the specificity and reversibility necessary to mimic the dynamic behavior of cellular membranes [12,13]. In addition, protein-mediated fusion in synthetic systems is frequently limited by challenges related to protein expression, stability, and integration into membranes [14].

**Figure 1:**
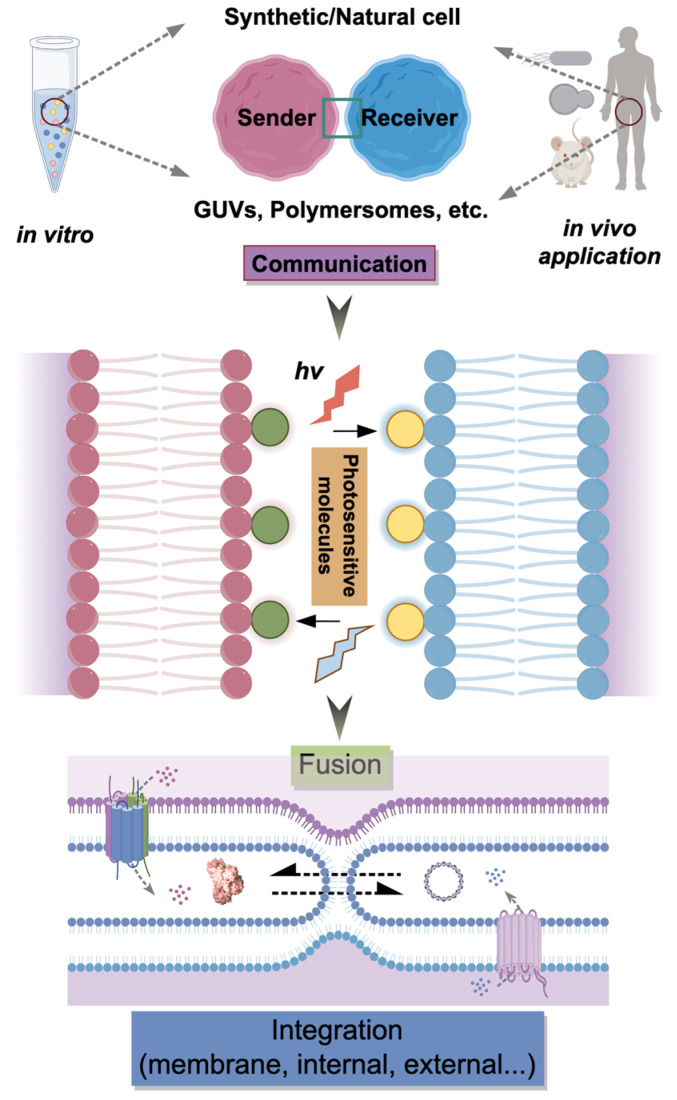
Photosensitive molecules enable designed communication, such as the member fusion events between synthetic (GUVs, polymersomes, etc.) and/or natural cells when precisely activated by different wavelengths (*hv*). To improve future biochemical, biomedical and clinical applications, the functionalized cells that come from both in vitro and in vivo models can be further optimized, for instance, by Design–Build–Test–Learn (DBTL) cycles. (Figure illustrated in Figdraw: https://www.figdraw.com, accessed on 7 January 2026).

These constraints necessitate alternative modalities that offer enhanced control over fusion dynamics. Light-induced membrane fusion has garnered significant attention due to its non-invasiveness, high spatiotemporal resolution, and tunability [12,15,16]. Light enables precise activation of fusion events at defined locations and times without chemical perturbations that could compromise system integrity. Recent advances demonstrate the feasibility of triggering fusion via specific light wavelengths, typically by incorporating photosensitive moieties [15,17,18] or tethered optogenetic protein modules [12]. The availability of light-triggered fusogens and their integration is rapidly expanding and has been excellently reviewed by Guinart et al. [12]. For instance, synthetic light-gated nanopores [15] and optogenetic protein dimerization systems [19] have been employed to regulate molecular transport and protein recruitment, providing a foundation for light-controlled membrane remodeling (Figure 1). Additionally, amphiphilic DNA nanostructures functionalized with hydrophobic anchors enable programmable adhesion and fusion of lipid vesicles [20,21,22], expanding the toolkit for light-responsive engineering.

Crucially, integrating light-induced fusion with synthetic cell platforms has yielded artificial cells with enhanced functional diversity. These systems dynamically assemble membraneless organelles [23], regulate enzymatic cascades, and modulate intracellular signaling pathways in a reversible, programmable manner [24,25]. This capability facilitates the design of synthetic organelle networks exhibiting adaptive feedback mechanisms and spatial reconfiguration [26], thereby emulating complex cellular behaviors. Such advancements deepen our understanding of fusion mechanisms and pave the way for innovative applications in drug delivery, biosensing, and regenerative medicine [4].

Despite promising developments, challenges remain in optimizing efficiency, specificity, and biocompatibility [8,27,28]. Translating these techniques to physiologically relevant conditions requires careful consideration of membrane composition, fusion kinetics, and cytotoxicity. Furthermore, integration with living cells should overcome barriers such as limited light penetration and immune responses [29]. Addressing these obstacles is critical for realizing the full potential of light-induced membrane fusion. In summary, the light-induced membrane fusion represents a transformative approach to synthetic cell construction, leveraging the precision of light to develop sophisticated systems mimicking dynamic cellular processes. We systematically summarize recent advances in light-mediated membrane fusion to highlight its role in synthetic cell engineering, with a focus on bottom-up synthetic biology and how these fusogenic synthetic cell can interface with biological entities through light-activated membrane fusion.

## 2. Light-Induced Membrane Fusion

### 2.1. Basic Principles and Mechanisms

#### 2.1.1. Design and Mechanism of Photosensitive Molecules

**Figure 2:**
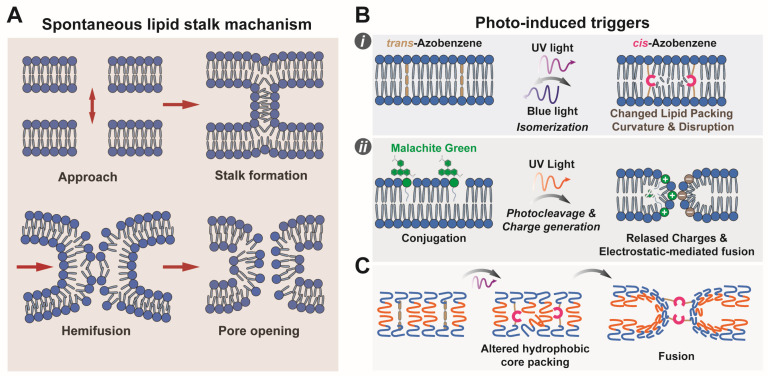
Photo-induced membrane fusion mechanisms by photosensitive molecules. (**A**) Stalk mechanism of membrane fusion. The sequential steps of membrane fusion are illustrated. First, the two membranes approach due to attractive potentials. They then deform to form a high-curvature stalk, which expands. This leads to a hemifused state, a metastable intermediate, that subsequently progresses to full fusion via pore opening in the adhering monolayers. (**B**) Photo-induced membrane fusion mediated by photosensitive amphiphiles. Two primary mechanisms are shown. *i*: Azobenzene isomerization: Azobenzene-containing amphiphiles switch between *trans* and *cis* isomers upon light exposure, altering the lipid packing parameter and inducing curvature strain in the membrane. *ii*: Photocleavage of Malachite Green moiety: UV light cleaves amphiphiles with a Malachite Green moiety, generating positively charged amphiphiles. These induce strong electrostatic interactions between membranes and modify amphiphile properties, promoting fusion. (**C**) Azobenzene-modified polymers: Polymers with azobenzene side groups undergo photoisomerization, changing the packing of polymer chains within the hydrophobic core of the membrane, thereby facilitating membrane fusion.

Photosensitive molecules drive light-induced membrane fusion by undergoing *trans* structural and electronic changes upon irradiation, subsequently inducing membrane remodeling (Figure 2). Common photosensitive moieties include azobenzene and acridone derivatives, each exhibiting distinct characteristics facilitating controlled membrane interactions [30]. The latter are photosensitizing molecules that promote side reactions with reactive oxygen species (ROS), such as singlet oxygen (^1^O_2_) [31,32], chemically altering the membrane and promoting fusion [33,34]. They offer little control over reaction dynamics and specificity, and induce non-reversible chemical alterations in the membrane. In azobenzene-containing amphiphiles, UV-induced *trans*-to-*cis* isomerization increases molecular curvature and packing defects within the lipid bilayer [17,18,31,32]. This destabilization lowers the energy barrier for fusion-stalk formation, facilitating the merging of adjacent membranes. Visible light irradiation restores the *trans* conformation, allowing reversible control over fusion dynamics. Other synthetic photoresponsive amphiphiles, such as malachite green (MG) derivatives and spiropyran (SP), have also been reported [35,36,37,38]. Malachite green is photocleaved by UV irradiation, yielding a soluble cyanide anion and leaving a positive charge on the lipophilic dye moiety, changing the amphiphilicity of the molecule and introducing charges that can result in attractive potentials with oppositely charged membranes, although the latter effect is small due to the small percentage of malachite green typically employed [36]. Spyropyrans convert to the merocyanine form reversibly by photoisomerization and show similar potential to azobenzene-containing systems, but have found narrower applications to membrane fusion up to now due to the large changes in lipid assembly they cause [39]. A visual representation of these membrane rearrangements in lipidic and polymer vesicles is provided in Figure 2.

Recent molecular design efforts focus on enhancing photosensitizer efficiency and specificity. Strategies include incorporating heavy atoms to promote ROS generation [40], tuning electronic structures to shift absorption into the near-infrared (NIR) region [41], and conjugating targeting ligands to localize photosensitizers [38]. For example, aggregation-induced emission (AIE) photosensitizers [42] utilize twisted conformations to suppress π-π stacking, enhancing ROS production and photostability. Additionally, supramolecular assemblies combining photosensitizers with peptide scaffolds [38] enable precise spatial organization and responsiveness. In summary, the design of photosensitive molecules leverages photoisomerization or photosensitization to induce structural changes or oxidative modifications. Ongoing research aims to optimize properties such as absorption wavelength, ROS yield, and biocompatibility to expand applicability in biological and therapeutic contexts.

#### 2.1.2. Advantages of Spatiotemporal Control in Light-Induced Membrane Fusion

Light-induced membrane fusion provides exceptional spatiotemporal control, which is critical for manipulating synthetic cells and functional biomimetic systems. As an external stimulus, light offers distinct spatial precision—down to subcellular or molecular scales—and temporal resolution ranging from milliseconds to minutes [15,16]. This precision is achieved via engineered photoreactive moieties or optogenetic modules that undergo conformational changes or binding state transitions upon illumination at specific wavelengths [43]. For instance, nucleic acid-functionalized membranes can initiate localized fusion events upon light-triggered activation of membrane-associated oligonucleotides [43]. Similarly, optogenetic dimerization systems, such as the improved Light-Inducible Dimer (iLID) [19], facilitate the selective recruitment of fusion mediators to defined subcellular regions, enabling localized fusion with micron-scale resolution and rapid kinetics. Tunable light parameters—intensity, wavelength, and duration—allow precise modulation of fusion efficiency. Their optimization requires balancing the photoconversion efficiency of the fusogen with the unwanted side-effects of prolonged exposure to high-intensity light. Most fusogens work in the UV spectrum, which might promote side reactions involving the fusogen itself or relevant biomolecules that may lose functionality. Higher intensities increase activation rates, while specific wavelengths ensure selective excitation without off-target effects. Furthermore, optimizing illumination duration balances cumulative activation against potential phototoxicity [43]. Crucially, light-controlled fusion can be reversible during the membrane fusion process; specific optogenetic systems [44] allow toggling between active and inactive states, enabling real-time modulation. Similarly, azobenzene-modified amphiphiles can reverse between *cis* and *trans* isomers by illumination with specific wavelengths, enabling photoswitching of membrane properties on short timescales [45]. Table 1 summarizes reported parameters for light-induced liposome fusion for several of the systems discussed here. Only a few examples of systematic optimization of photoconversion parameters were identified [46], indicating the need for a more thorough reporting on the optimization procedures for these systems. For example, reported values for irradiation power usually refer to the power of the source, not necessarily the irradiance at the sample. This indicates the need for more systemic and comparative studies to ensure reproducible results and reporting, and to establish photoinduced fusion as a reliable technique for microcompartment manipulation.

The precision of light-mediated fusion is advantageous for constructing compartmentalized synthetic cells, where fusion orchestrates cargo delivery, enzymatic cascades, or signaling pathways. Unlike chemical inducers limited by diffusion and irreversibility, light offers a superior modality for in vivo and complex tissue applications. Membrane fusion with high spatial and temporal control is the subject of a large body of research aimed at enhancing liposome-based drug delivery platforms. Magnetic fields [50] and ultrasound [51,52] are under active exploration for this purpose. Both offer a lower theoretical spatial resolution compared to light, especially taking into account non-linear optical effects exploited in multiphoton absorption. Nevertheless, their enhanced tissue penetration (several mm to whole organ/body, compared to sub-mm of UV-visible light and a few mm for NIR in biologically compatible windows) is appealing. Light remains a preferential avenue for triggered fusion in vitro since it can be trivially coupled to available light microscopy techniques, enabling concomitant stimulation and observation [48], which requires more specialized equipment for magnetic or ultrasonic stimuli. Ultimately, integrating light-responsive elements into fusion systems affords unparalleled spatiotemporal control, advancing the functionalization and programmability of synthetic cellular constructs [19,43,44].

#### 2.1.3. Classification and Comparison of Existing Light-Induced Membrane Fusion Techniques

Light-induced membrane fusion techniques fall into two primary categories: chemical photosensitizer-triggered methods and optogenetic tools. Chemical photosensitizers typically employ exogenous molecules that generate reactive species upon activation to induce membrane destabilization [12,31,46]. While these methods offer rapid response times and ease of implementation, they often lack spatial precision and carry cytotoxicity risks due to reactive intermediates. Conversely, optogenetic approaches utilize genetically encoded proteins to control fusion with high spatiotemporal resolution, enabling precise manipulation within living or synthetic systems [19,44,53]. However, these techniques require complex genetic engineering and typically exhibit slower kinetics than chemical methods. Recent hybrid strategies combining photosensitizers with optogenetic modules [54] have demonstrated enhanced fusion efficiency with minimized adverse effects. For example, integrating channel rhodopsin-based actuators with localized photosensitizers improves control over fusion sites while reducing off-target damage [55]. Selection of the appropriate strategy depends on balancing application-specific requirements, including fusion speed, spatial precision, biocompatibility, and system complexity.

### 2.2. Light-Induced Membrane Fusion in Liposomes and Polymersomes

#### 2.2.1. Structural Characteristics and Functional Advantages of Polymer Vesicles

Polymersomes (polymer vesicles) are self-assembled hollow nanostructures formed by amphiphilic block copolymers, mimicking the architecture of natural cell membranes [24,56]. Driven by amphiphilicity, hydrophobic and hydrophilic segments spontaneously organize in aqueous environments to minimize free energy, creating bilayered structures [57]. This self-assembly process is highly tunable; variations in polymer composition, molecular weight, and block ratio allow precise control over vesicle size, membrane thickness, and permeability. For instance, thermoresponsive amphiphilic graft copolymers can form unilamellar vesicles (40–70 nm) via temperature cycling, demonstrating structural memory and reversible monomer-vesicle transitions [58]. Furthermore, membrane thickness is adjustable—ranging from 52 Å in pure lipid vesicles to 97 Å in pure polymer vesicles—by varying copolymer molecular weights or creating lipid-hybrid systems [59]. Functionally, polymer vesicles offer robust membranes, overcoming the mechanical instability and rapid degradation typical of lipid vesicles [60]. They exhibit enhanced mechanical strength, reduced permeability to small molecules, and prolonged circulation times, making them superior candidates for drug delivery [60] and synthetic cell construction [61]. Their membranes can be engineered for semi-permeability [62] or to incorporate functional nanoparticles [63] and biomolecules, imparting capabilities such as antimicrobial activity [64], photothermal responsiveness [65], or catalysis [61]. This chemical versatility allows the design of stimuli-responsive elements, enabling controlled release or remodeling via pH, temperature, or light triggers [65,66]. In essence, polymer vesicles combine the structural robustness of synthetic polymers with the functional mimicry of biological membranes, positioning them as essential tools for constructing stable, multifunctional synthetic compartments. Polymersomes’ tunable structural robustness comes at the cost of biomimetic flexibility [24]. Their thicker membranes are less flexible and thus less prone to structural rearrangement through membrane fusion. Additionally, tri-block co-polymers are bola amphiphiles that form membranes with a distinct architecture compared to the lipid bilayer, to which the stalk model of membrane fusion might not apply. These factors might explain the paucity of reported works on light-induced fusion on polymersomes. We highlight here current approaches to triggered polymersome fusion, to assess novel approaches to photoinduced polymersome fusion.

#### 2.2.2. Recent Advances in Triggered Polymer Vesicle Fusion

Triggered fusion of polymer vesicles is a critical research area in synthetic cell construction [67], offering a pathway to dynamically control compartmentalization and content mixing. Recent studies have elucidated the mechanisms of chemical and light-induced fusion, advancing our understanding of molecular events and enabling precise manipulation of synthetic assemblies. Chemical triggers, such as metal ion coordination [68] or pH modulation [69,70], exploit changes in membrane charge, hydration, and packing to overcome the inherent stability that renders polymer vesicles resistant to spontaneous fusion [67]. While direct examples of light-induced polymer vesicle fusion are still limited, insights from protein vesicle systems—where thermoresponsive polypeptides coupled with light-activated crosslinking drive fusion [71]—suggest that integrating photo-cross-linkable moieties [43] can achieve comparable spatiotemporal control. To dissect these processes, time-resolved small-angle X-ray scattering (TR-SAXS) provides real-time structural data on vesicle morphology and fusion intermediates [72,73]. For example, TR-SAXS combined with dissipative particle dynamics (DPD) simulations has mapped fusion pathways in star block terpolymer vesicles, identifying stages such as pore formation and hemifusion [73]. Complementary techniques like quartz crystal microbalance (QCM-D) and atomic force microscopy (AFM) [70] further clarify how polymer block length and membrane composition influence fusion kinetics. These multi-modal analyses reveal that while shorter hydrophobic blocks facilitate restructuring, longer blocks enhance robustness at the cost of fusion propensity [70]. Collectively, these advances enable the rational design of dynamic synthetic compartments capable of controlled cargo delivery and membrane remodeling.

#### 2.2.3. Application Potential of Light-Induced Polymer Vesicle Fusion

Light-induced polymer vesicle fusion is a promising frontier for intelligent drug delivery, nanoreactors, and synthetic cell systems. By combining the stability and tunability of polymer vesicles [60,66] with the non-invasive precision of light, this approach enables targeted cargo release and compartmental reorganization. In drug delivery, light-induced fusion facilitates on-demand release of therapeutic agents [65,74], minimizing systemic side effects. Triggering fusion at specific tissue sites ensures drugs are released precisely, overcoming premature degradation [51]. Beyond delivery, this technology enables the construction of nanoreactors—artificial compartments that mimic cellular organelles [62]. Fusion events can modulate internal environments by mixing substrates or enzymes, driving complex reaction cascades within confined spaces [61]. In synthetic cell systems [5,61], light-induced fusion emulates behaviors such as membrane remodeling and vesicle trafficking (Figure 2C). For instance, synthetic protein condensates that recruit and release proteins upon light stimulation [44,75] exemplify how optogenetic control can manipulate phase-separated compartments. Such programmable soft materials [76] pave the way for tailor-made synthetic cells [77]. Consequently, light-induced polymer vesicle fusion has the potential to advance the integration of artificial constructs with living systems and may contribute to future developments in medicine and fundamental cell science [1,78,79].

### 2.3. Application of Light-Induced Membrane Fusion in Functionalization of Synthetic Cell Membranes

#### 2.3.1. Membrane Protein Functional Reconstitution and Optically Controlled Fusion Techniques

Membrane proteins are pivotal in synthetic cell membranes, mediating signaling, transport, and fusion. Functional reconstitution of these proteins is critical for constructing biomimetic systems [14,80]. Optical control of membrane fusion offers high spatiotemporal precision in modulating protein activity and localization [14]. Optogenetic tools are increasingly used to induce protein–protein interactions or conformational changes upon illumination [44]. For example, optogenetic manipulation of syntaxin clusters regulates synaptic SNARE complex assembly, elucidating fusion mechanisms in live cells [53]. Similarly, dimerization systems like iLID enable the targeted recruitment of proteins to specific membrane regions [19], regulating signaling networks with subcellular resolution. The efficacy of these strategies depends on optimizing membrane anchoring domains and fusion configurations. Furthermore, light-gated tools incorporating BAR domains (e.g., CRY-BARs) exploit intrinsic membrane-binding properties to actively remodel membrane architectures [81]. Optogenetics also permits dynamic regulation of lipid signaling molecules, such as phosphoinositides [82], and heterotrimeric G-proteins [83], which influence trafficking and fusion. Mechanistically, light-induced fusion regulates protein localization and activity by facilitating controlled complex assembly, altering curvature, and modulating lipid composition [84]. This rapid, reversible control allows for the dissection of physiological processes like synaptic vesicle exocytosis [85] and organelle remodeling [86], advancing the functional reconstitution of membrane protein networks in synthetic cells.

#### 2.3.2. Molecular Transport and Signal Transduction via Light-Induced Membrane Fusion in Synthetic Membrane Systems

Light-induced membrane fusion is a highly controllable strategy for mediating molecular transport and signal transduction within synthetic systems [1]. By harnessing light as a trigger, fusion events can be precisely timed and spatially regulated [43], enabling the selective exchange of proteins, nucleic acids, or signaling compounds between compartmentalized synthetic cells. This mimics natural cellular communication, where fusion facilitates cargo delivery and signal propagation [79]. Recent advances show that light-inducible synthetic protein condensates [44] can dynamically recruit or release proteins in living cells, effectively controlling downstream signaling cascades [75,87]. In synthetic systems, light-induced fusion facilitates the targeted exchange of signaling molecules across membranes, orchestrating communication within synthetic cell communities [1,78]. The reversible nature of these events allows for temporal control of signal transduction, essential for mimicking dynamic biological networks [88,89]. Engineering controlled molecular exchange holds significant promise for constructing sophisticated synthetic cell consortia that exhibit coordinated behaviors [1], adaptive responses, and programmable functionalities [5,90]. Thus, light-induced membrane fusion is a promising approach for building synthetic networks with integrated molecular communication, which could facilitate the development of functional synthetic tissues.

#### 2.3.3. Light-Induced Membrane Fusion Facilitates Interactions Between Synthetic Cells and Natural Cells

Light-controlled membrane fusion enables direct interactions between synthetic and natural cells [91] by precisely triggering lipid bilayer merging [92]. The design of synthetic cells with specifically engineered biological responsivity enables the use of compartmentalized systems beyond simple cargo delivery, and instead as properly interactive therapeutics that can self-regulate to provide adaptive therapeutic strategies [4]. In this context, photoinduced membrane fusion between biological and synthetic cells promises to integrate these capabilities with excellent spatiotemporal control over cell–cell interactions. Synthetic cells can be engineered with photosensitive fusogenic components, such as light-responsive peptides [93] or nucleic acid conjugates [91,94], which promote destabilization and fusion with target cells upon illumination [95]. This facilitates the exchange of molecular contents, enabling functional integration with living systems [96,97]. For example, lipidated coiled-coil peptides mediate efficient fusion between synthetic liposomes and natural membranes [97], enhancing the intracellular delivery of therapeutics like doxorubicin. In cell therapy, this direct membrane merging [98] bypasses endocytic pathways [99,100], thereby reducing lysosomal cargo degradation and improving therapeutic efficacy [101,102]. Furthermore, spatial regulation allows for targeted activation at specific tissue sites [74], minimizing off-target effects. Synthetic membranes mimicking natural fluidity, such as zwitterionic dendrimersomes [103], further facilitate this integration. In biosensing, fusion events can create hybrid systems that respond to environmental signals [104,105] or initiate synthetic signaling pathways within natural cells [79]. Despite these prospects, challenges remain in fusion efficiency and specificity, requiring systematic studies that up to now have concerned few photoresponsive fusogens [93,106]. Furthermore, their biocompatibility requires thorough investigation in vivo, representing one of the main challenges to address. Insights from viral fusion mechanisms [107] and the development of less immunogenic membrane-active peptides [93] are critical for optimizing these interactions for clinical translation.

### 2.4. Recent Advances and Challenges in Light-Induced Membrane Fusion Technology

#### 2.4.1. Advanced Photo-Controlled Materials and Technological Innovations

Novel photosensitive materials have significantly advanced light-induced membrane fusion, particularly for synthetic cell functionalization. Notably, synthetic protein condensates [44,75] can dynamically recruit or release proteins in living cells via light-controlled phase separation [75,87]. Integrating optogenetic tools with these condensates [54,108] allows reversible protein sequestration [75,109], permitting the manipulation of processes like membrane ruffling within minutes [44]. Parallel technological progress in light delivery has been crucial for improving efficacy. Multiphoton excitation, which utilizes simultaneous absorption of lower-energy photons, allows activation at greater depths with reduced tissue damage [40], facilitating in vivo applications. Similarly, near-infrared (NIR) responsive materials [41,110] leverage superior tissue penetration and minimal phototoxicity to trigger fusion non-invasively [74]. These advances enhance biocompatibility and expand potential clinical applications, such as targeted drug delivery. Together, integrating innovative materials with sophisticated light activation techniques advances the design of functional synthetic cells.

#### 2.4.2. Major Challenges and Solutions in Light-Induced Membrane Fusion Technology

Despite its precise spatiotemporal control, light-induced membrane fusion technology faces critical challenges in efficiency, selectivity, and biosafety. Fusion efficiency is often limited by the heterogeneous expression of optogenetic components and protein diffusion, which reduces spatial precision [19,97]. Furthermore, selectivity is compromised by potential off-target interactions [95] and the difficulty of restricting fusion to specific membrane domains [94], particularly in carrier systems like extracellular vesicles [96,106]. Biosafety is a primary concern for in vivo applications; UV or high-intensity light can induce phototoxicity and oxidative stress [32,43], while immunogenic fusion proteins may provoke adverse immune responses [111]. To address these limitations, optogenetic tools with optimized anchoring domains have been engineered to enhance local recruitment and reduce diffusion [19,53]. Strategies to mitigate phototoxicity include developing actuators responsive to longer wavelengths (e.g., NIR) [41,110] and utilizing photopharmacological agents requiring minimal light doses [112]. Additionally, coupling light activation with pH-sensitive linkers or fusogenic lipids [98] can facilitate fusion under milder conditions. Finally, multivalent targeting probes on nanoparticles [94,111] can improve specificity. A multifaceted approach combining molecular engineering, optimized light parameters, and complementary biochemical strategies is essential for advancing safe, effective synthetic cell systems.

#### 2.4.3. Interdisciplinary Integration Driving Advances in Light-Induced Membrane Fusion

The advancement of light-induced membrane fusion relies on the integration of materials science, bioengineering, and optical technologies [113]. Materials science contributes novel photoswitchable lipids and hybrid membranes [17,114], such as azobenzene-based molecules that induce fusion via reversible isomerization. Bioengineering harnesses the molecular recognition of nucleic acids [43] to functionalize membranes [91], enabling light-activated control over cell-like compartments and catalytic cascades [61,113]. Meanwhile, optical technologies provide high-resolution control and monitoring. Innovations like Light–Oxygen–Voltage (LOV) domains fused to membrane-targeting proteins allow for subcellular manipulation of organelle membranes [86], while optimized recruitment systems [19,53] enhance fusion kinetics. Converging these disciplines—from molecular design to optical control—is essential for overcoming limitations and realizing the full potential of complex, functional synthetic cells.

## 3. Conclusions and Outlook

Light-induced membrane fusion has emerged as a transformative technique for assembling and functionalizing synthetic cells. By leveraging photosensitive molecules and optogenetic tools, this methodology enables precise spatiotemporal regulation of membrane merging, which is superior to conventional chemical or thermal triggers (Figure 1). This capability is instrumental for orchestrating complex molecular events, including cargo delivery [51], enzymatic cascade assembly [61], and inter-compartmental communication [1]. However, challenges regarding fusion efficiency and phototoxicity persist [43,97]. Future research must prioritize materials operating with red or near-infrared (NIR) light for in vivo utility [41,74] and the creation of hybrid systems responsive to multiple stimuli (e.g., pH, temperature) to enhance adaptability [67]. Additionally, a deeper mechanistic understanding of membrane remodeling via advanced techniques like TR-SAXS [73] is required to rationally design next-generation fusogens. Continued innovation in this domain will accelerate the translation of synthetic cells toward viable biomedical applications [29,96], including intelligent drug delivery, biosensing, and tissue engineering [115].

## Figures and Tables

**Table 1:** Comparison of reported photoinduced membrane fusion parameters and efficiencies.

Reference	Photosensitive Molecule	Source	Power	Wavelength	Time	Quantification Method	Fusion Efficiency (%)
[35]	Malachite Green (2%)	Xe lamp	500 W	<300 nm	15 min	SUVs size increase	NA
[47]	PolyAzoPAM (block co-polymer)	Hg lamp	100 W	365 nm	3.5 s	Individual events monitored by microscopy	NA
[17]	AzoPC (20–27 mol%)	UV lamp	8 W	365 nm	7 min	Lipid mixing assay	20–27%
[36]	Malachite Green (2%)	Xe lamp	500 W	300–400 nm	5 min	ANTS quenching content exchange assay	10–15%
[36]	AzoTAB (0–100 mol%)	Tunable light source	0–100 μW	365 nm	0–30 min	Imaging flow cytometry content exchange assay	60%
[48]	AzoTAB (50 mol%)	Hg lamp	15–30 μW	330–385 nm	1 s	Individual events monitored by microscopy	NA
[49]	Malachite Green (0–2.5 mol%)	Xe lamp	500 W	300–400 nm	10 s	Endosome escape and leakage measurement	NA

## Data Availability

No new data were created or analyzed in this study. Data sharing is not applicable to this article.

## Abbreviations

The following abbreviations are used in this manuscript:

GUVs	Giant unilamellar vesicles
SNAREs	Soluble NSF attachment protein receptors
ROS	Reactive oxygen species
MG	Malachite green
SP	Spiropyran
AIE	Aggregation-induced emission
NIR	Near-infrared
iLID	Improved light-inducible dimer
TR-SAXS	Time-resolved small-angle X-ray scattering
DPD	Dissipative particle dynamics
QCM-D	Quartz crystal microbalance
AFM	Atomic force microscopy
LOV	Light–oxygen–voltage
DBTL	Design–Build–Test–Learn
